# Exploring the Antecedents to the Reputation of Chinese Public Sector Organizations During COVID-19: An Extension of Situational Crisis Communication Theory

**DOI:** 10.3389/fpsyg.2022.818939

**Published:** 2022-06-13

**Authors:** Zhao Chunxia, Wang Fei, Fang Wei

**Affiliations:** ^1^School of Business Administration, Hebei Normal University of Science and Technology, Qinhuangdao, China; ^2^Theoretical Economics Post-Doctorate Research Station, School of Chemistry and Environmental Engineering, Shenzhen University, Shenzhen, China; ^3^International Business School, University of International Business and Economic, Beijing, China

**Keywords:** crisis responsibility, crisis response strategy, internal crisis communication, organizational reputation, China

## Abstract

The study is intended to examine the impact of crisis responsibility on the reputation of the Chinese public sector organization during the COVID-19 crisis. In addition to that, the study has also examined the mediating role of crisis response strategy in the relationship between crisis responsibility and the reputation of the Chinese public sector organization during the COVID-19 crisis. Lastly, the study has also examined the moderating role of internal crisis communication in the relationship between the crisis responsibility and crisis response strategy of the Chinese public sector organization during COVID-19. This study bases on a quantitative research approach along with a cross-sectional research design. The population of the study is the Chinese public sector organization companies. Data is collected from the employees working in Chinese public sector organizations. The current study used 500 sample sizes; 205 valid responses are received which were used in data analysis. The SEM-PLS is used for the data analysis. The result of the study indicates that all the direct hypotheses are accepted significantly, which indicates that the crisis responsibility and crisis response strategy are significant determinants of organizational reputation during the COVID-19. In a similar vein, the crisis responsibility has placed a significant impact on the crisis response strategy of Chinese organizations during COVID-19. The study is among the pioneer studies on crisis responsibility in the Chinese public sector organization during COVID-19. The study has employed an extended framework encircling the literature on crisis responsibility, crisis response strategy, internal crisis communication, and organizational reputation. Theoretically, the study has extended the situational crisis communication theory framework by incorporating internal crisis communication in the framework.

## Introduction

The reputation management area has been gaining increased interest among scholars to examine criticism regarding reputation management as well as issues that affect the reputation of public organizations. The challenges and issues during COVID-19 associated with reputation management are public safety, situations which can have a direct impact on the life of citizens, for instance, public policies, and general elections which affect the public and are generally based on the public’s trust in the organization during COVID-19 (Luoma-aho and Canel, 2020).

Since the emergence of coronavirus (COVID-19), there have been increasing levels of stress, uncertainty, and emotional disruption around the world. Even organizations and businesses are trying hard to overcome the challenges associated with the pandemic by providing resources to their stakeholders. However, businesses need effective communication to develop trust among the stakeholders through practicing empathy and transparency. Effective crisis response strategy facilitates organizations and businesses in developing hope and trust among the stakeholders and in adapting to the faced challenges during COVID-19. The world has never witnessed such a crisis in the past, therefore, keeping in view the current situation, the communicators and leaders are making efforts to formulate a plan for crisis communication, with an aim to connect communities, stakeholders, and the employees. In this regard, a course of action must be carefully chosen while developing such a plan, as it is required to be informative, effective, and appropriate for the given situation.

The literature on crisis responsibility analyzed and limited research and studies were found concerning the favorable public sector reputation (in context to the benefits and its implications) in comparison to the private sector reputation. Although there is a lack of studies in this area, scholars and researchers have been showing conducting studies to examine the causes of the crisis in public sector organizations and their potential consequences. Christensen and Lægreid (2020) study reported that crises negatively influence the public sector organizations’ reputation. They further argued that with the increasing emergence of crises, researchers have been taking more interest in exploring how these crises affect organizational reputation. Furthermore, with the increased globalization, the causes and the treatment of the crisis have been changing. Therefore, firms are required to incorporate international learning for effective crisis management as the local strategies now seem less effective.

Reputation management awareness has been significantly improved since the public organization-related incidents have been increasing rapidly. As a result, COVID-19, scholars also realized that just like private sector firms, public organization management is also important since it affects the reputation and survival of the public organization (Busuioc and Lodge, 2017; Pyrozhenko, 2020).

Since there is a lack of theory-based research available, therefore there is a need to extend explanatory theories focusing on the indicators and determinants of organizational reputation and crisis. Several scholars have attempted to present a theoretical framework for the organizational crisis, for instance, Benoit (2020) presented the image repair theory, Tao et al. (2021) situational theory of publics, and Scherer (2018) put forward the attribution theory. An integrated four-step symmetrical model is another theory that explains the organizational crisis (Aljuhmani and Emeagwali, 2017). Even though all these models have successfully explained the organizational crisis, they had some limitations.

For instance, the attribution theory by Martinko and Mackey (2019) successfully established an empirical link between organizational reputation and crisis. However, it failed to provide leaders with a guiding principle whenever a crisis occurs. On the other hand, the image repair theory also ignored a key element used in the phase of crisis management. It only presents a theoretical application but failed to analyze multiple issues that may affect the organization’s image restoring process (Almarshoodi, 2021).

Therefore, this study aims to use Dotson’s (2017) situational crisis communication theory (SCCT) to develop an understanding of the victim type of the crisis to the preventable type by considering the case of a non-employee’s death incident as a preventable crisis. The study also aims to examine crisis response strategies that the organization will employ and how they will impact the organizational reputation.

Prior studies (Islam and Ali, 2018; Higgins, 2020) on crisis communication and organizational reputation show that internal communication drives the crisis response perception both directly and indirectly which thus leads the public to perceive or assume organizational reputation (Bridgewater, 2021). Communication plays a key role during the process of crisis response strategy selection since it aids in maintaining or restoring organizational identity and reputation (Islam and Ali, 2018; Antwi, 2021). Internal communication is considered an important variable that directly or indirectly affects the relationship between organizational reputation ad crisis communication (Burrell et al., 2018; Burrell, 2019). Thus, during a crisis, poor internal communication may cause issues for the organization as the information that comes from the organization forms the basis for public perceptions. Therefore, this information directly or indirectly influences the attitudes of the public toward the organization. During the organizational crisis, internal communication has some implications for the management and business practices which have not been tested empirically and theoretically (Andersson, 2019). Therefore, the study has planned to achieve the following research objectives:

• To examine the impact of crisis responsibility on the reputation of the public sector organizations in China during COVID-19.
• To examine the mediating role of crisis response strategy in the relationship between the crisis responsibility and reputation of the public sector organizations in China during COVID-19.
• To examine the moderating role of internal crisis communication in the relationship between the crisis responsibility and crisis response strategy of the public sector organizations in China during COVID-19.

## Literature Review

### Organizational Reputation

Despite the extensive discussion and studies regarding organizational reputation, this concept is still controversial and debatable. Most of the research on this is confined to the private sector which resulted in the lack of studies on public sector organizations’ reputation, and it remained an underused resource (Esen et al., 2021). For instance, issues such as the benefits of reputation, its constraints and restrictions, challenges, characteristic differences, and how to handle constraints and challenges remained overlooked and have not been recognized clearly.

Several prior studies on public organizations have been examined and found that not enough studies have been conducted regarding the public organization’s reputation in context to reputational perspective. Most of the studies were mainly performed by focusing on the public organizational issues that have an indirect impact on the organization’s reputation. Thus, the reasoning behind this rationale is that public organization-related issues form the basis for their reputation among the public.

For instance, some of the researchers also attempted to focus public perception on the public service quality, democratic ethos, public sector management (Beeri, 2021), performance management of public administration (Raharjo and Eriksson, 2017; Altamimi and Jimenez, 2021), perception about performance and politics, and public trust and perception on government (Beeri, 2021). All these studies have not mentioned it explicitly that all organizational outcomes such as reputations do have their implications. Technically, the public organization’s reputation is not presented in context to legislation rather stakeholders’ perception toward public organizations is formed based on the performance of the organization (NawoseIng’ollan and Roussel, 2017).

Although, organizational reputation is a controversial concept because there has been a lack of consensus on the definition and approach of this concept, such as constructs, dimensions, and measurement issues (Kim and Woo, 2019; Hasan and Hossain, 2021), political sensitivity, threats and crisis, drivers that influence the public perception of good reputation, reputation’s financial value, relation with leadership commitment, and performance management (Halligan, 2021), and influence issues. The academic literature has failed to provide a clear connection of public organizations’ reputations.

It has now become a priority for public organizations to create and maintain a good reputation (Canel et al., 2020), however, the public organizations’ intrinsic political nature often creates restrictions for the organizations to maintain excellent reputation (Aher and Luoma-Aho, 2017). Such political constraints can be in the form of political pressures and issues that affect the consistency of reputation, restrict the freedom of organizations in strategic matters, and affect the organization’s charismatic ability to handle respective stakeholders. These restrictions not only make it difficult for the organization to establish excellent public perception toward their reputation but even more to maintain it.

### Crisis Responsibility

The literature has been thoroughly examined to examine the crisis responsibility connection in public organizations, but little evidence has been found about this attribution. A general discussion on organizational crisis responsibility will be done in this section by analyzing and presenting examples of popular case studies. The Exxon Valdez oil disaster (EVOS) 1989 is a popular case of the largest spill that took place in coastal waters in the US. In the oil and gas industry, it is also recognized as an environmental tragedy that occurred due to human error (Theophilus et al., 2017). Although, the company fully claimed the responsibility for the incident and shifted its focus on its restoration and remediation, this incident severely damaged the reputation of Exxon Corporation.

Similarly, in the oil and gas industry, two big names also disappeared upon refusal for taking responsibility for the incident. At the beginning of 2001, the Enron scandal led the Enron Corporation toward bankruptcy. In addition, the well-known accounting and auditing firm not only faced a downfall but also lost Arthur Andersen. A simple fraud in accounting caused irregularities that greatly affected the reputation of Arthur Andersen (Akpanuko and Umoren, 2018).

Delaying the crisis responsibility process increases the threat to the organization’s reputation as the delay will cause the media to assume that the organization is planning to cover up. Portraying an organization as responsible and at fault also damages the organization’s reputation (Etter et al., 2019). Similarly, if the organization takes the responsibility for the crisis, it is assumed that the organization admits that this crisis is caused by its actions and is then held responsible to manage the crisis. Contrarily, if the organization does not take responsibility and denies it, then it is perceived that an outside factor or another party is involved and is responsible for the crisis. Prior research shows that the potential reputational threat to the organization reduces when the organization takes the responsibility while the organization’s reputation is severely damaged if it denies taking the responsibility.

### Crisis Response Strategy

The crisis response strategy refers to the approach that is followed by the organization during the crisis (Nekmat and Kong, 2019). According to Nekmat and Kong (2019), an effective crisis response strategy comprises three components. These are:

1. Crisis response strategy is effective if it provides the organization members with the information that could help them in offsetting the damage caused by the crisis.
2. Establishing a crisis response strategy using the available information about crisis management and causes and types of the crisis.
3. An effective crisis response strategy should consider the reasons and causes of the crisis and must be conveyed to the concerned stakeholders in a timely manner.

The crisis response strategy aims to mainly focus on the situation of the crisis and not on the reputation (Nekmat and Kong, 2019). In SCCT, the attribution theory explains the connection of crisis type with crisis response strategy. Martinko and Mackey (2019) argued that attribution theory emphasizes that individuals should accept the responsibility of all events either favorable or unfavorable and must take necessary actions based on the information provided to them. The attribution theory presents principles for the individuals to understand, anticipate, and evaluate events against any unforeseen occurrences (Nekmat and Kong, 2019).

According to the SCCT model, crisis response strategy significantly influences crisis responsibility. There is still a dearth of studies concerning the significance of crisis response strategy in crisis responsibility and crisis management (Vafeiadis et al., 2019). The issue of public response was explained by a study in which public perception toward Samsung was discussed in context to SCCT. The study argued that in a situation when the provided information is differentiable at low levels, then it increases the tendency among people to make higher internal attribution or lower external attribution. In another study, Vafeiadis et al. (2019) studied crisis management by applying the SCCT theory to the case of the American Red Cross. According to the findings of this study, developing an effective crisis response strategy can be useful in effectively handling the crisis. During a crisis, an organization’s stability is associated with the organization’s prior reputation. It suggests that when a threat appears to the organization’s reputation, then the firm has to choose a crisis response strategy. This theory is advantageous as it examines the reactions and perceptions of the stakeholders toward the crisis.

### Internal Crisis Communication

It is observed that most prior studies have emphasized mainly external communication, and the significance of internal communication has only been acknowledged until recently (Adamu and Mohamad, 2019; Mazzei and Butera, 2021). According to the prior studies, the most relevant concept to prevent the crisis is internal communication. Internal communication will likely affect the culture, relationships, and climate of the organization which can also influence organizational changes, and their implementation (Adamu and Mohamad, 2019). Besides, it also facilitates handling workers’ responses and reactions. Since employees effectively reflect and advocate the company’s communication strategies and its reputation, therefore, any negative reaction or communication by the employee may affect the reputation of the company (Verčič, 2021). Internal communication serves as a guiding force that supports reactions, controls damage, prevents the occurrence of crisis, and brings about positive outcomes.

All such factors negatively influence organizational credibility which is a key factor in crisis management. Therefore, organizational members are required to investigate the crisis using their internal links and sources. Considering the situation, there might be the possibility that mass media gets access to the internal information and in that case, the management of the organization should be well aware of how to effectively, and accurately establish strong communication with the members of the organization to execute the planned process. Internal crisis communication refers to the effective and useful process of collaboration among internal stakeholders and managers at any stage i.e., prior, post, or during COVID-19 as well as in any type of organization, be it public, private, or NGOs.

Adamu et al. (2018) defined ICC as the communication condition of an organization when an unfortunate incident occurs and develops a connection of trust among both parties by establishing two-way communication by including the internal stakeholders. Several internal stakeholders’-based studies reported that students are also a form of human capital and are a key asset for educational institutions.

### Organizational Reputation During COVID-19

Governments around the world have been facing massive challenges resulting from COVID-19, ranging from income support to generating strong health services, to providing support to the companies who are struggling. Besides, a remarkable amount of collaboration and cooperation is also required among the nations on successfully obtaining vaccines and testing and detecting the virus. The ability of a state to handle such a crisis depends on how much cumulative investment it has made on its management and governing ability. Countries that overlook the need to make investments in dynamic capabilities face serious challenges and issues when such a COVID-19 crisis hits them (Amankwah-Amoah et al., 2021).

Before COVID-19, the main concerns of most governments were to deal with wicked issues and challenges faced by the modern world, such as demographic challenges, promoting the people’s well-being, and climate change (Spinelli et al., 2020). Such challenges also involve the struggle to generate an inclusive and sustainable level of growth in the economy. Currently, policymakers have diverted their attention toward the rate and direction of economic growth (Spinelli et al., 2020). Regenerating public and private investments, collaboration and innovation are the essential elements that are required to deal with the current challenges. A state is assumed to be capable of acting as a first resort, i.e., crowding in investments (private), and stimulating growth and innovation in potential growth areas. It also demands a unique collaboration among the business and the state and not picking winners rather the ones who are willing to collaborate (Singh et al., 2021).

The outbreak of the novel coronavirus has accelerated and intensified the need to develop a challenge-based policy framework. The current pandemic situation provides policymakers and governments with the opportunity to critically analyze their policy foundations and observe areas that need realignment of policy and resources with the twenty-first century needs. The global pandemic also emphasized the significance of capabilities and capacity of the public sector, as it is expected to be capable to deal with emergencies and the required capabilities that are needed for resolving societal issues and challenges, such as, public health. Thus, the COVID-19 pandemic emphasized public sector both as a market fixer and the market shaper for the economy (Dwianto, 2021).

## Hypothesis Development

According to the SCCT model, a crisis’s reputational threat should be considered for determining the appropriate crisis response strategy (Nekmat and Kong, 2019). Nekmat and Kong (2019) also argued that reputation threat is one of the keys determining factors in choosing the right strategy. The model suggests that initial crisis responsibility is determined by framing the crisis and its type. Once the crisis responsibility is assigned, the next step is to analyze the crisis management history and examine the case where other companies or current companies have faced a similar situation in past. The aim of choosing the crisis response strategy should not emphasize the reputation or its reconditioning rather on the crisis. Attribution theory explains the connection between the crisis type and crisis response in SCCT. According to Chernyak and Tziner (2016), the attribution theory explains the key role of an individual in claiming self-responsibility of the event either unfavorable or favorable. The theory advocates that it is the responsibility of the individual to take required actions based on the information available to him. The attribution theory presents principles for the individuals to understand, anticipate, and evaluate events against any unforeseen occurrences (Nekmat and Kong, 2019) and to cope with them. A crisis response strategy is mainly determined by the organization’s prior reputation (Nekmat and Kong, 2019). During a crisis, an organization’s stability is associated with the organization’s prior reputation. It suggests that when a threat appears to the organization’s reputation, then a firm is required to own the responsibility of crisis. According to the literature Schaedler et al. (2021), organizational reputation is significantly influenced by crisis responsibility. As crisis responsibility is attributed based on the type of the crisis, therefore, such attributions then affect the organization’s reputation, and the impact on the organization varies as the attribution of crisis responsibility varies (Nekmat and Kong, 2019). As the crisis responsibility attribution increases so do the reputational threat to the organization. The SCCT proposes that crisis types, crisis response strategy, and attribution of crisis responsibility are directly associated with each other. It is assumed that crisis type shapes the way crisis responsibility is attributed and determines the attitude of the organization i.e., accommodative, or defensive toward the crisis. The greater the responsibility assigned the more accommodative an organization would be. Therefore, the reputational threat to the organization and its severity depends on the crisis responsibility perception. Thus, we propose the following hypothesis:

***H1:*** *Crisis Responsibility has a significant impact on the reputation of public sector organizations during COVID-19.*

According to the literature, organizational reputation is significantly and directly influenced by crisis responsibility (Nekmat and Kong, 2019). As crisis responsibility is attributed based on the type of the crisis, such attributions then affect the organizational reputation, and its impact on the organizations varies as the attribution of crisis responsibility varies (Nekmat and Kong, 2019). As the crisis responsibility attribution increases so do the reputational threat to the organization. SCCT proposes that crisis types, crisis response strategy, and attribution of crisis responsibility are directly associated with each other. It is assumed that crisis type shapes the way crisis responsibility is attributed and determines the attitude of the organization i.e., accommodative, or defensive toward the crisis. The greater the responsibility assigned the more accommodative an organization would be. Thus, the reputational threat to an organization and its severity depends on the crisis responsibility perception.

The crisis responsibility is attributed to the stakeholders considering the reasons and potential causes of the crisis which triggered the situation (Jarim, 2017). Initial crisis responsibility explains that to what extent stakeholders assume the organization is responsible for the crisis, and thus reflects how they perceive the situation and the organization. According to the prior literature (Nekmat and Kong, 2019), a negative relationship exists between crisis responsibility attribution and favorable organizational reputation. The greater the crisis responsibility assigned to the organization, the more damage it will cause to the organizational reputation. Similarly, if the organization under question is a reputed organization, then the organization has to face more scrutiny and media will likely create a sensation out of it (Jamilah, 2016). Zheng et al. (2018) studies reported that positive organizational reputation minimizes the severity of the threat posed by the crisis. The study also proposed that the impact of the crisis weakens when the organization has a good reputation and vice versa.

The findings obtained in Zheng et al. (2018) study support and are in favor of strong reputed organizations, and also explain that the level of attributed crisis responsibility indicates how much communication efforts are needed by the organization. Thus, the more the crisis responsibility assigned to the organization the greater will be the efforts that will be needed for rebuilding the organizational reputation.

A thorough review of the literature suggests that the strategic actions of the organization i.e., to reject or accept the responsibility of the crisis play a significant role in restoring the damaged reputation resulting from the crisis threat (Nekmat and Kong, 2019). Given scholars (Schaedler et al., 2021), an organization’s reputation and its willingness to take crisis responsibility are directly related. Generally, organizations are assumed to be responsible for the occurrence of any event or crisis and claiming crisis responsibility by the organization leads to a positive impact on its reputation. Consistently, researchers (Nekmat and Kong, 2019; Schaedler et al., 2021) suggest that there is a need to conduct evidence-based research to confirm whether the organization’s decision to accept or to reject the assigned responsibility is valid or not. The cause of the crisis must be identified by the organization through crisis framing to determine the type of the crisis, besides, the crisis responsibility must also be attributed before applying any crisis response strategy.

***H2:*** *Crisis response strategy has a significant impact on the reputation of public sector organizations during COVID-19.*

***H3:*** *Crisis response strategy mediates the relationship between the crisis responsibility and the reputation of public sector organizations during COVID-19.*

Waqas et al. (2017) defined the term communication as a process of acquiring, creating, distributing, integrating, or applying the knowledge with an aim of developing capabilities that would lead the firm toward better organizational performance resulting from collective as well as individual learning. Such as it would be relatively easier for the competitors to imitate individual learning, whereas it would be difficult for them to imitate a collective and continuous organizational learning process (Sidani and Reese, 2018). Internal communication is considered an essential component for the organization’s smooth day-to-day functioning, while effective crisis management, plays an extensive role. Thus, it can be concluded that during a crisis, inadequate, untimely, inaccurate, or delayed information significantly impact the employees’ perception and their trust toward the organization which affects their level of commitment toward the organization in crisis resolution. During a crisis, inaccurate information is one of the greatest threats to the organization as it leads to the emergence of rumors and defeatist declarations. Reputation-based research put forward situations can affect the perception of the stakeholders toward organizational reputation. In view of Nekmat and Kong (2019), the perceptions of the stakeholders can be shaped by crisis framing, which will influence the stakeholders’ perception and how they perceive the crisis. Nekmat and Kong (2019) argued that the prior reputation of the organization and the crisis history both indirectly and directly influence the organizational reputation in a crisis. Although, no empirical evidence is found concerning the potential threat that crisis responsibility attribution poses on the organizational reputation. According to Zheng et al. (2018), the stakeholders’ perception of the consequences of the crisis affects how they react to the situation assuming that the crisis will have both indirect and direct impacts on them. In this way, organizations get affected by the crisis and face apparent consequences, such as, damages, breakdowns, victims, losses, change in goals, strategies, and purpose of the organization, or poses threat to the structure of the organization, these consequences appear as a result of media trial and because of the lack of communication among the members which negatively affects the efficiency, intervening capability, and morale of the personnel.

***H4:*** *Internal crisis communication moderates the relationship between the crisis responsibility and Crisis response strategy of public sector organizations during COVID-19*.

## Methodology

The current study is survey-based, and a questionnaire survey is used. The relationship between crises responsibility, organizational reputation, crisis response strategy, and internal crisis communication matter is examined with the help of a questionnaire. Primary data is collected through survey questionnaires which were adapted from prior studies. Therefore, this study is based on a quantitative research approach along with a cross-sectional research design. The population of the study is the Chinese public sector organization during COVID-19. Data is collected from the employees working in the public sector organization of Hubei Province of China during COVID-19.

The questionnaire is designed by using the scale items revealed in previous studies. Crises responsibility is measured by using six scale items, organizational reputation is measured by using 15 scale items, crisis response strategy is measured by using six scale items, and finally, five items are used to measure internal crisis communication matter. Therefore, all the measures are adapted from previous studies and designed a survey questionnaire which is based on two major sections. The first section is based on the respondent’s profile including age, education, marital status, income, and designation. Furthermore, the sample size is selected based on previous studies. Various studies in the concerned field used 300–500 sample sizes. Thus, the current study used 500 sample sizes. The total usable responses received were, which further processed for the analysis. Additionally, data statistics are given in Table 1 showing the missing value and also reporting outliers as well as determining the normality of data.

**TABLE 1:** Data statistics.

	No.	Missing	Mean	Median	Min	Max	SD	Kurtosis	Skewness
CR1	1	0	1.96	2	1	5	0.94	1.17	1.108
CR2	2	0	2.047	2	1	5	1.25	0.544	1.245
CR3	3	0	1.846	2	1	5	0.903	1.571	1.251
CR4	4	0	1.772	2	1	5	0.913	1.617	1.325
CR5	5	0	2.114	2	1	5	1.251	0.417	1.175
CR6	6	0	2.027	2	1	5	1.117	1.17	1.289
OR1	7	0	1.826	2	1	5	0.939	2.528	1.535
OR2	8	0	1.872	2	1	5	1.032	1.281	1.297
OR3	9	0	1.98	2	1	5	1.006	1.799	1.317
OR4	10	0	2.007	2	1	5	1.19	0.961	1.339
OR5	11	0	2.054	2	1	5	1.299	0.482	1.272
OR6	12	0	1.919	2	1	5	1.102	1.267	1.346
OR7	13	0	1.926	2	1	5	1.075	0.818	1.164
OR8	14	0	1.966	2	1	5	1.058	1.49	1.372
OR9	15	0	1.846	2	1	5	0.925	1.508	1.29
OR10	16	0	2.06	2	1	5	1.119	0.838	1.187
OR11	17	0	1.913	2	1	5	1.086	1.084	1.317
OR12	18	0	1.987	2	1	5	1.176	1.106	1.353
CRS1	19	0	1.826	2	1	5	1.008	2.174	1.509
CRS2	20	0	2.027	2	1	5	1.042	0.802	1.131
CRS3	21	0	2.201	2	1	5	1.153	–0.026	0.9
CRS4	22	0	1.752	2	1	5	0.919	3.555	1.721
CRS5	23	0	2.034	2	1	5	1.144	0.934	1.267
CRS6	24	0	2.054	2	1	5	1.14	0.471	1.103
ICCM1	25	0	2.027	2	1	5	1.135	0.934	1.227
ICCM2	26	0	2.255	2	1	5	1.301	–0.267	0.918
ICCM3	27	0	2.221	2	1	5	1.247	–0.294	0.893
ICCM4	28	0	2.356	2	1	5	1.326	–0.794	0.647
ICCM5	29	0	2.383	2	1	5	1.288	–0.637	0.72

## Data Analysis

**FIGURE 1:**
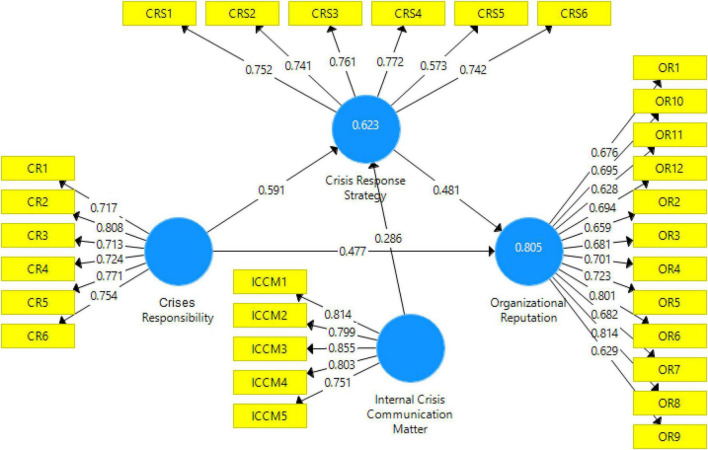
Measurement model.

**FIGURE 2:**
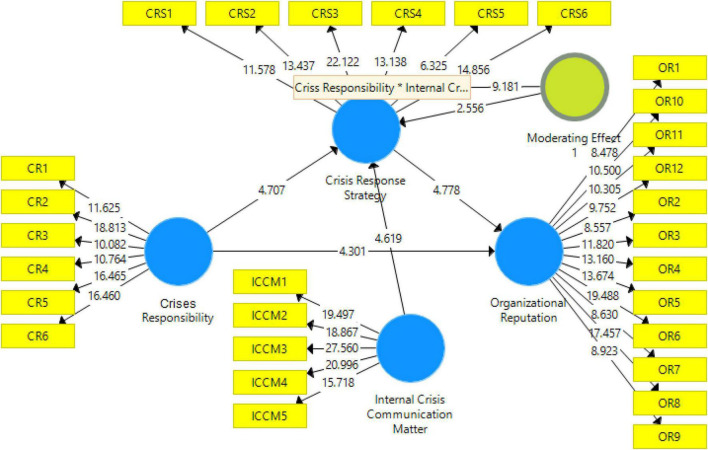
Structural model.

Data analysis of the current study is based on PLS-SEM. PLS-SEM is the most recommended data analysis technique which is recommended by several studies as the statistical analysis used in social sciences (Hair et al., 2016, 2017). PLS-SEM is majorly based on two steps: measurement model and structural model. PLS structural model is given in Figure 1 in which the confirmatory factor analysis (CFA) is carried out. The main purpose of the CFA is to determine the reliability (factor loading, CR, AVE) and validity of the model, This study considered 0.6 as the minimum threshold level to retain the scale items. Results in Figure 2 show that the threshold value of item loading is maintained at 0.60 and any item with loading less than 0.60 is excluded from the final model. Furthermore, to confirm the convergent validity, this study examined CR and AVE. Both values are reported in Table 2. CR must be above 0.7 and AVE must be above 0.5. Results show that; all the constructs have CR above 0.7 and AVE above 0.5 which confirmed the convergent validity. Finally, discriminant validity (Henseler et al., 2015; Sarstedt et al., 2020) is examined by using the Heterotrait-monotrait Ratio of Correlations (HTMT). HTMT_0_._9_ indicates that none of the values should be higher than 0.9. It is given in Table 3.

**TABLE 2:** Factor loadings, composite reliability (CR), and convergent validity.

Variables	Items	Loadings	Alpha	CR	AVE
Crisis responsibility	CR1	0.717	0.843	0.884	0.56
	CR2	0.808			
	CR3	0.713			
	CR4	0.724			
	CR5	0.771			
	CR6	0.754			
Crisis response strategy	CRS1	0.752	0.819	0.869	0.528
	CRS2	0.741			
	CRS3	0.761			
	CRS4	0.772			
	CRS5	0.573			
	CRS6	0.742			
Internal crisis communication matter	ICCM1	0.814	0.864	0.902	0.648
	ICCM2	0.799			
	ICCM3	0.855			
	ICCM4	0.803			
	ICCM5	0.751			
Organizational reputation	OR1	0.676	0.905	0.92	0.501
	OR10	0.695			
	OR11	0.628			
	OR12	0.694			
	OR2	0.659			
	OR3	0.681			
	OR4	0.701			
	OR5	0.723			
	OR6	0.801			
	OR7	0.682			
	OR8	0.814			
	OR9	0.629			

**TABLE 3:** HTML.

	Crisis response strategy	Crisis responsibility	Internal crisis communication matter	Organizational reputation
Crisis response strategy				
Crisis responsibility	0.731			
Internal crisis communication matter	0.727	0.653		
Organizational reputation	0.773	0.844	0.664	

**FIGURE 3:**
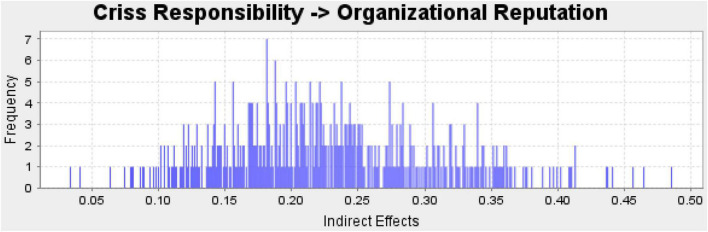
Indirect effect histogram.

The second step of PLS-SEM is PLS structural model which is used to examine the relationship between variables. In this step, PLS bootstrapping is used which is most recommended by previous studies (Hair et al., 2013, 2014; García-Fernández et al., 2018; Nuseir et al., 2020; Basheer et al., 2021; Raoof et al., 2021). This study considered *t*-value 1.96 as the minimum threshold level to accept the hypotheses. In the current study, all the hypotheses are supported because all the hypotheses have a *t*-value above 1.96. Direct in effect results are given Table 4 along with the moderating effect. The indirect effect is given in Table 5. Additionally, an indirect effect histogram is presented in Figure 3.

**TABLE 4:** Direct effect results.

	(O)	(M)	(STDEV)	(| O/STDEV|)	*P*-values
Crisis response strategy → Organizational reputation	0.481	0.492	0.101	4.778	0
Crises responsibility → Crisis response strategy	0.463	0.455	0.098	4.707	0
Crises responsibility → Organizational reputation	0.477	0.467	0.111	4.301	0
Internal crisis communication matter → Crisis response strategy	0.29	0.307	0.063	4.619	0
Moderating effect 1 → Crisis response strategy	0.124	0.123	0.049	2.556	0.011

*O, Original sample; M, Sample mean; STDEV, Standard deviation; |O/STDEV|, T Statistics.*

**TABLE 5:** Indirect effect results.

	(O)	(M)	(STDEV)	(|O/STDEV|)	Values
Crises responsibility → Crisis response strategy → Organizational reputation	0.223	0.227	0.078	2.845	0.005

*O, Original sample; M, Sample mean; STDEV, Standard deviation; |O/STDEV|, T Statistics.*

The results of the study indicate that all the direct hypotheses are accepted significantly, which indicates that the crisis responsibility and crisis response strategy are significant determinants of organizational reputation during the COVID-19. In a similar vein, the crisis responsibility has placed a significant impact on the crisis response strategy of Chinese organizations during COVID-19 a low crisis responsibility is attributed to the organization in case of victim cluster. In an intentional or preventable crisis, strong crisis responsibility is attributed to the organization. Meanwhile, the effectiveness of the implemented strategies cannot merely be assessed using SCCT theory as this theory focuses only on the crisis type and response strategy and how they affect the organizational reputation. In context to organizational communication, several factors affect the organizational outcomes during a crisis. These factors also include factors internal to the organization, such as crisis responsibility, and leadership communication.

The crisis response strategy significantly mediates the relationship between the Crises Responsibility—Organizational Reputation.

**FIGURE 4:**
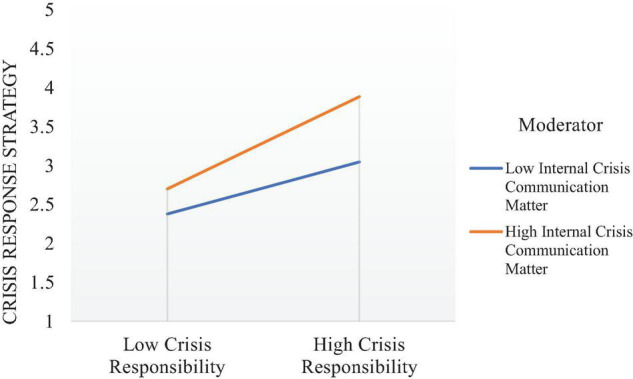
Moderation effect.

Finally, this study examined the moderation effect of internal crisis communication matter between crisis responsibility and crisis response strategy. This effect is shown in Figure 4 which indicates that; internal crisis communication matter strengthens the relationship between crises responsibility and crisis response strategy. The results of interaction of internal crisis communication in the relationship between the crisis response strategy and crisis responsibility are also significant.

Furthermore, the r-square value is reported in Figure 1 which is 0.805. This r-square value is strong (Hair et al., 2019; Shehzadi et al., 2020) which shows that all the variables are expected to bring an 80.5% change in organizational reputation.

## Conclusion and Discussion

Based on the above-mentioned results, we assume that a responsible organization tends to accept the responsibility whenever any crisis appears and tries to take corrective measures for crisis management. Most of the time, the actions of the organization depend largely on who and what caused the crisis. In the crisis management process, the first step is always to frame the crisis by exploring the type of the crisis. The crisis responsibility during COVID-19 helps in identifying who should be attributed as responsible for the crisis. The crisis responsibility during COVID-19 is attributed based on the crisis framing, and its type, such as intentional, accidental, or victim clusters.

The prior studies (Kiersch and Peters, 2017; Azizah et al., 2021) reported a direct linkage of organizational reputation and communication. In this research, we aim to extend the SCCT theory through integrating organizational credibility and leadership communication to explore the reputation of the public organization during a crisis of COVID-19. The process of internal communication usually comprises idiosyncratic individual, who interacts and collaborates with other individuals through knowledge and information exchange. To codify and break down the tacit knowledge into organizational capital or tangible processes a system should be developed by the firm. Thus, efforts must be made by the organization to develop an internal communication management system. Internal communication is a dynamic process for creating social and human capital for the organization which may lead the firm toward sustainable competitiveness (Zhu et al., 2018). Financial performance is the key success indicator in private organizations and competition exists between them, whereas public organizations gain benefits by establishing a good reputation and by protecting it. For this purpose, bureaucratic firms, for instance, regulatory bodies, ministries, and local and federal agencies work and collaborate to maintain a favorable reputation. Thus, public organizations are required to consistently protect and nurture their reputation.

The result of the study offers unique implications for managers and in particular employees of public sector organizations aimed COVID-19. By incorporating crisis response, crisis response strategy, and internal crisis communication, the study has extended the normal scope of crisis management during COVID-19 by offering new insights into SCCT in the public organization China. The extended model coupled with the new insights offered in studies will equip the managers with tools for managing crisis. The practical contributions of the study are multifold.

The findings of the study have revealed that the conceptualization of internal crisis communication in unique settings of China is different from the western settings as the study has conceptualized the leader as a man of crisis with powerful oration skills and command of language and formal communication. The study not only considers the communication, but also has taken into account the CST.

## Limitations and Future Directions

The study has worked on the public sector organizations in a given situation. The results of the study are limited in their application. The findings cannot be applied to any other sector or organizations experiencing a different crisis. Similarly, the respondents of China have been targeted in the study, which narrows down the scope. The results can differ for any other part of the economy. In other countries, the public sector organizations may have a difference in values, which can yield different results. Thus, the outcomes of this study cannot be applied to any other sector or organization. The organizations possessing similar values in any other sector or economy can take advantage of the findings of this study, as the public sector organizations in Asian countries have many resemblances with the public sector of China.

## Data Availability Statement

The original contributions presented in this study are included in the article/supplementary material, further inquiries can be directed to the corresponding author.

## Ethics Statement

Ethical review and approval was not required for the study on human participants in accordance with the local legislation and institutional requirements. Written informed consent for participation was not required for this study in accordance with the national legislation and the institutional requirements.

## Author Contributions

All authors listed have made a substantial, direct, and intellectual contribution to the work, and approved it for publication.

## Conflict of Interest

The authors declare that the research was conducted in the absence of any commercial or financial relationships that could be construed as a potential conflict of interest.

## Publisher’s Note

All claims expressed in this article are solely those of the authors and do not necessarily represent those of their affiliated organizations, or those of the publisher, the editors and the reviewers. Any product that may be evaluated in this article, or claim that may be made by its manufacturer, is not guaranteed or endorsed by the publisher.
